# Mobility of Carriers in Strong Inversion Layers Associated with Threshold Voltage for Gated Transistors

**DOI:** 10.3390/mi16121393

**Published:** 2025-12-09

**Authors:** Hsin-Chia Yang, Sung-Ching Chi, Bo-Hao Huang, Tung-Cheng Lai, Han-Ya Yang

**Affiliations:** 1 Department of Electronic Engineering, Ming Hsin University of Science and Technology, Hsinchu County 30401, Taiwan; chisc@must.edu.tw (S.-C.C.); loliconboss0@gmail.com (B.-H.H.); l3406549@gmail.com (T.-C.L.); 2 Department of Electronic Physics, National Yang-Ming Chiao-Tung University, Hsinchu City 300, Taiwan; rebeccaxd29@yahoo.com.tw

**Keywords:** MOSFET, FinFET, IGBT, threshold voltage, radiation by accelerated charges, strong inversion layer, kink effects, quantum confinement

## Abstract

NMOSFET, whose gate is on the top of the n-p-n junction with gate oxide in between, is called the n-channel transistor. This bipolar junction underneath the gate oxide may provide an n-n-n-conductive channel as the gate is applied with a positive bias over the threshold voltage (V_th_). Conceptually, the definition of an n-type or p-type semiconductor depends on whether the corresponding Fermi energy is higher or lower than the intrinsic Fermi energy, respectively. The positive bias applied to the gate would bend down the intrinsic Fermi energy until it is lower than the original p-type Fermi energy, which means that the p-type becomes strongly inverted to become an n-type. First, the thickness of the inversion layer is derived and presented in a planar 40 nm MOSFET, a 3D 240 nm FinFET, and a power discrete IGBT, with the help of the p (1/m^3^) of the p-type semiconductor. Different ways of finding p (1/m^3^) are, thus, proposed to resolve the strong inversion layers. Secondly, the conventional formulas, including the triode region and saturation region, are already modified, especially in the triode region from a continuity point of view. The modified formulas then become necessary and available for fitting the measured characteristic curves at different applied gate voltages. Nevertheless, they work well but not well enough. Thirdly, the electromagnetic wave (EM wave) generated from accelerating carriers (radiation by accelerated charges, such as synchrotron radiation) is proposed to demonstrate phonon scattering, which is responsible for the Source–Drain current reduction at the adjoining of the triode region and saturation region. This consideration of reduction makes the fitting more perfect. Fourthly, the strongly inverted layer may be formed but not conductive. The existing trapping would stop carriers from moving (nearly no mobility, μ) unless the applied gate bias is over the threshold voltage. The quantum confinement addressing the quantum well, which traps the carriers, is to be estimated.

## 1. Introduction

### 1.1. Modified Conventional Formula and Model

**Figure 1:**
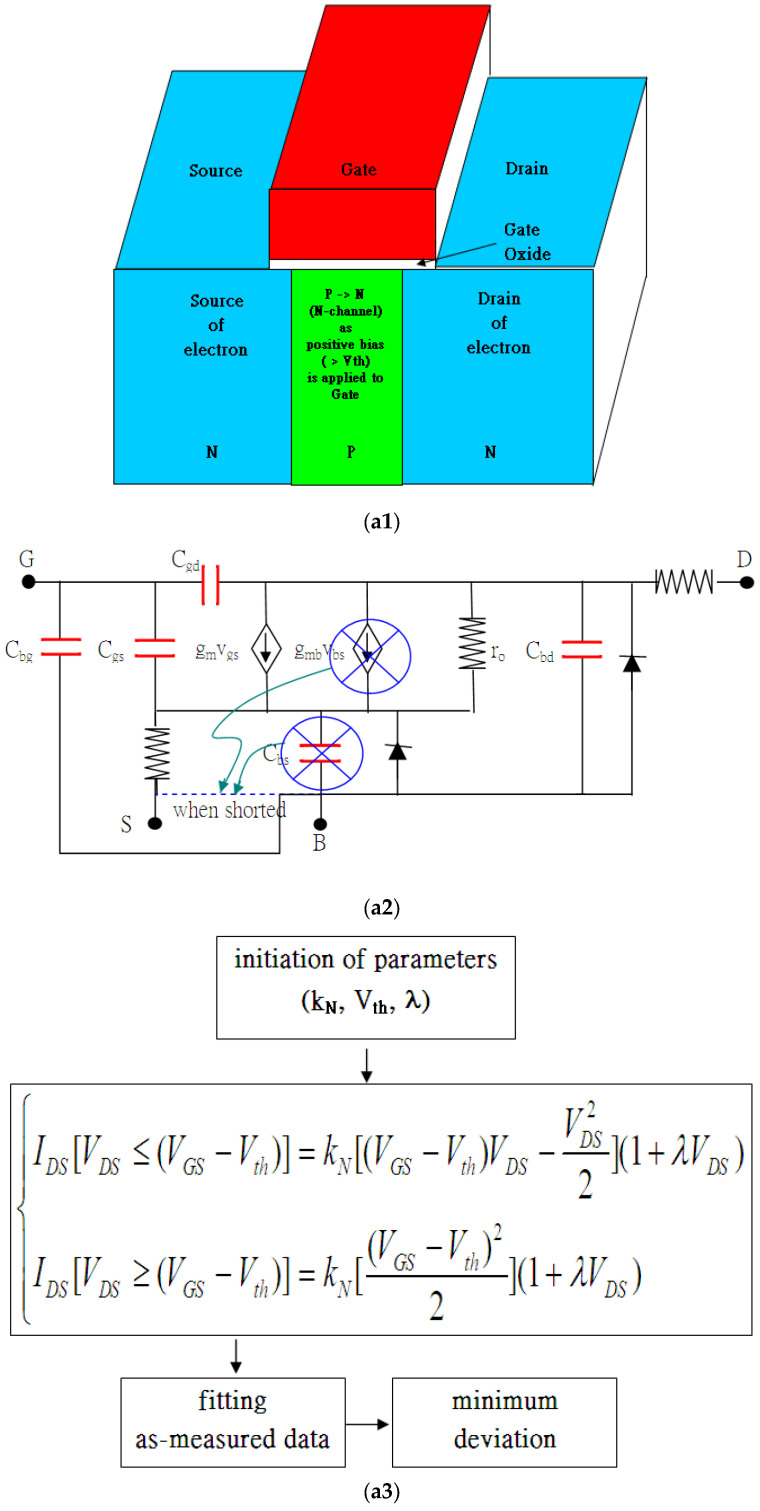

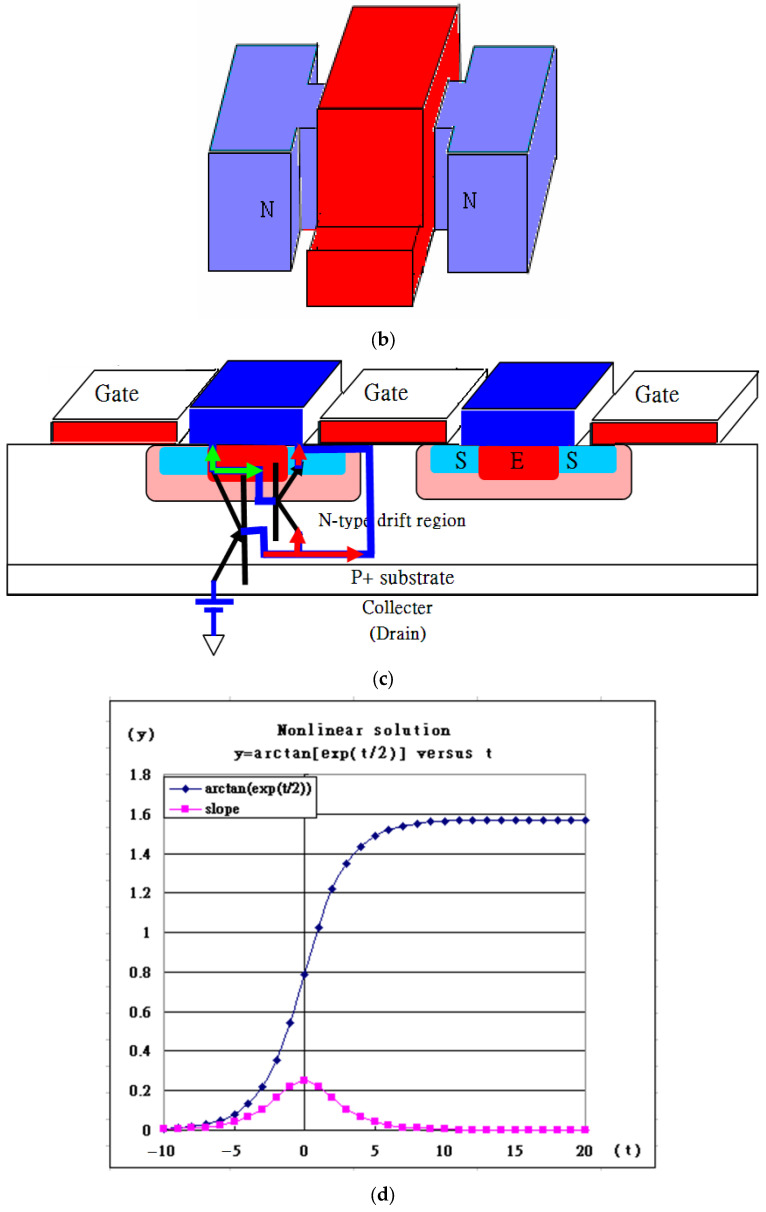
(**a1–a3**) NMOSFET cross section and the equivalent circuits. (**b**) The overview of FinFET transforming from NMOSFET in (**a1–a3**), whose gate extends downward to enclose the channel. (**c**) The n-p-n-p-equivalent current flow in IGBT cross section. (**d**) Nonlinear solution y = arctan[exp(t/2)] versus t looks like I_DS_-V_DS_ characteristic curves with slope variations. The kink causes heat dissipation problems because the slope maximum is equivalent to the speed maximum variation (acceleration) that causes extra heat. Acceleration radiation or braking radiation is then taken into account.

This study is based on the mechanism that, in NMOSFET (transistors using electrons as carriers for signals or power), n-p-n may become n-n-n-conductive due to the strong inversion layer. Conventional current–voltage formulas for this transistor use (k_N_, V_th_, λ) as the basic parameters to further build up a sophisticated model like BSIM4 (simulation manual generated by UC Berkeley). BSIM4 facilitates itself with many equivalent circuits and, thus, introduces many other physical parameters, e.g., as shown in Figure 1(a1–a3). In Figure 1(a2), ***r_o_*** (***I_o_***λ***V_DS_****** = V_DS_/r_o_***) is added to address the leakage current between the Drain and the Source in the saturation regime. Somehow, the triode regime shares the equivalent r_o_ as well. From the continuous point of view, the two proposed formulas, including the triode regime and saturation regime, are supposed to be continuous at V_DS_ = (V_GS_ − V_th_). Therefore, the conventional formulas modified by adding (1 + λV_DS_) in the triode regime are mandatory. On the other hand, the term (1 + λV_DS_) does address the leakage current and shall be reasonably taken into account in the triode region as well [1]. Somehow, V_th_ may vary as the short-channel effect and drain-induced barrier lowering are considered. As in Figure 1(a2), some equivalent capacitors, such as C_gs_ or C_gd_, show open circuits unless the input frequencies are high enough. Also, g_m_ (trans-conductance) plays a crucial role, as observed in the slope of I_DS_-V_GS_, which shows the optimization of an amplifier on the voltage gain. Many other parameters include G_m_ in the slope of I_DS_-V_DS_ characteristics, making equivalent passive devices for the feasibly continuous feature. As the two-port network is considered in Figure 1(a2), the h_21_ (short-circuit current gain) and S_22_ (output reflection coefficient, g_m_) are comparatively considered, where kinks are found [2].

In a circuit design, Kirchoff’s current law and Kirchoff’s voltage law mainly address the whole circuit design with passive devices, which obey the generalized Ohm’s law (*v = iZ_X_, X = R, L, C*), and active devices, whose characteristics are characterized by models. Currently, the BSIM model starts with a MOSFET of a large length (L_o_) and width (W_o_) to extract V_tho_ by fitting the as-measured data without short/narrow channel effects. Then, two sets of MOSFET with a smaller fixed L or W are proposed. One is a set of fixed L (L < L_o_) with (L, W_1_,), (L, W_2_), (L, W_3_), …or (L, W_MIN_), and the other is a set of fixed W (W < W_o_) with (L_1_, W), (L_2_, W), (L_3_, W), …or (L_MIN,_ W). Both sets of transistors are measured, fitted, and extracted as a model. This generated model may address all transistors of various sizes in the circuit. With computer aid, the circuit is evaluated at every node and at every branch. Any node converges with a voltage and any branch with a current.

As for the extraction of the parameters in this paper, a MOSFET and an FinFET of a specific channel length and channel width are taken into account, whose as-measured data is fitted with the modified conventional formula. Based on the deviation of the minimum root mean square, parameters (k_N_, V_th_, λ) are extracted. Intriguingly, some extracted parameters, such as the mobility in k_N_, may reveal interesting underlying physics.

### 1.2. Isolated Gate Bipolar Transistor

Unlike traditional planar MOSFET, 3D FinFET and Isolated Gate Bipolar Transistor (IGBT) (Figure 1) use the same gated control mechanism. Three-dimensional FinFET is popularly applicable because the channel is highly depleted. The depletion region always reduces the leakage current effectively, even as the channel length is shrinking (Figure 1b). IGBT may equivalently refer to the structure combination of the isolated gate-controlled MOSFET and Bipolar Junction Transistor (BJT). The equivalent BJT structure in the IGBT may provide a comparatively high current gain. Also, there exist insulated gates, beneath which double diffusions of Arsenic and Boron simultaneously undertake at high temperature to form an equivalent N-channel MOSFET. Such a double-diffusion NMOSFET structure is completely the same as in the traditional power MOSFET. Uniquely, extra heavily doped Boron in the central parts is surrounded by heavily doped Arsenic in nearby gates, as shown in Figure 1c. The current controlled by the insulated gate triggers BJTs in the equivalent circuit, which turn on via the applied bias on the gates. There are two types of BJT: NPN and PNP. The P-type semiconductor in the middle of the NPN BJT is the triggering Base, and the other two N-type ones are the Emitter/Collector. On the other hand, the N-type semiconductor in the middle of the PNP BJT is also triggering the Base, and the other two P-types are the Emitter/Collector. The I_CE_ current of the BJT is associated with the Base current by either inputting or extracting the current, and it enjoys a comparatively high current gain, as in many other similar applications [3,4,5,6,7,8,9,10,11,12,13,14,15,16,17,18].

### 1.3. Kink Effect

Meanwhile, in order that the fitting may be further improved, the kink effects are then introduced to address the elimination of I_DS_ due to obstacles from phonons that may be coming from accelerating carriers (radiation by accelerated charges). As is known, each applied Source–Drain bias corresponds to an internal terminal speed of the carriers and, thus, a Source–Drain current, I_DS_, which may reach a maximum in the saturation region if there is no leakage current. The carriers are fermions, whose wave function may be expressed as follows:

$$\Psi_{e}=\sum_{\alpha }\phi_{e\text{\_}\alpha }\psi_{f\text{\_}\alpha },$$

where $\psi_{f}$ obeys Fermi–Dirac statistics. In addition, $\phi_{e\text{\_}\alpha }$ is the solution of a sine-Gordon equation, presented as follows:

$$\phi_{e}(x,t)=f_{e}(x,vt)=f_{e}(\xi )$$

and

$$\begin{array}{l} L=\frac{1}{2}(\frac{\partial \phi_{e}}{c\partial t}{)}^{2}-\frac{1}{2}(\frac{\partial \phi_{e}}{\partial x}{)}^{2}-V(\phi_{e})\\ where\\ V(\phi_{e})=\frac{a}{b}(1-\cos b\phi_{e})\\ and\\ \frac{\partial }{\partial t}\frac{\partial L}{\partial (\frac{\partial \phi_{e}}{\partial t})}+\frac{\partial }{\partial x}\frac{\partial L}{\partial (\frac{\partial \phi_{e}}{\partial x})}-\frac{\partial L}{\partial \phi_{e}}=0 \end{array}$$

where b may be (1/(5.43 Å) and φ is the scale around 5.43 Å, which corresponds to the carrier speed around v > 6.62 × 10^−34^/(5.43 × 10^−10^)/(9.11 × 10^−31^)(m/s) = 1.34 × 10^7^ m/s (Uncertainty Principle, *Δp > h/Δx)* such that *J = nev* > (1 × 10^22^) (1.6 × 10^−19^ )(1.34 × 10^7^) > 10^10^ (A/m^2^) and I > 10^10^ (A/m^2^) (10^−7^ × 10^−8^ m^2^) = 10^−5^ A. The scale estimation is indeed feasible.

If a=1=b, for simplicity,

$$\frac{\partial^{2}\phi_{e}}{\partial \xi^{2}}-\sin\phi_{e}=0$$

with the solution *f_e_(ξ) = 4 tan^−1^(ξ)*. The radiation is proportional to the slope of the current, i.e., the accelerating carriers, which are charged. The slope of the solution is calculated as follows:

$$\frac{df_{e}(\xi )}{d\xi }=\frac{4}{e^{\xi }+e^{-\xi }}=\frac{4}{2+\xi^{2}}\to 2\exp(-\frac{\xi^{2}}{2}),$$

which approaches Gaussian, and so is the EM radiation [19,20,21]. The maximum speed of the current always happens at the saturation regime, while the maximum variation occurs somewhere in between V_DS_ = 0 and V_DS_ = (V_GS_ − V_th_). The maximum variation in the speed of the carriers corresponds to the maximum variation in *J = nev*. It, thus, radiates a maximum EM wave, agitating more lattice vibrations (quantized as phonons), which result in more phonon scattering. Phonon scattering is, thus, responsible for the Source–Drain current reduction at around V_DS_ = (V_GS_ − V_th_) [19,20,21]. The kinks are defined to be the maximum deviation in the fitting data and the as-measured data and are almost linearly proportional to V_DS_ = (V_GS_ − V_th_). Indeed, the reliable model must be based on the fitting with minimum deviations, and the root mean square really helps. The generated model is, thus, facilitated to design an integrated circuit (IC).

### 1.4. Various Possibilities of Kinks

I_DS_-V_DS_ and I_DS_-V_GS_ in planar MOSFET and 3D FinFET commonly enjoy the same two formulas in Figure 1(a1–a3), where both G_m_ and g_m_ do demonstrate kinks of their own kinds. As shown in Figure 1d, kinks make the circuit design achieve specific optimum purposes, e.g., an amplifier with high voltage gain (S_22_). As for the IGBT, equivalent BJT-like n-p-n and p-n-p are structurally shown everywhere. Therefore, the current gain (current gain can be distributed by I_Base_ or V_CE_; *h_21_* defines the contribution from I_Base_ alone) is, thus, stressed, and the kink of h_21_ may be found in the following:

$$h_{21}\equiv {\left(\frac{\partial I_{CE}}{\partial I_{Base}}\right)}_{V_{GE}=fixed}$$

In this paper, three transistors with gated control are presented, including a 40 nm MOSFET, L240 W120 FinFET, and square gated IGBT. For the IGBT, the authors focus on those biases applied to the insulated gate, which is slightly larger than the internal threshold voltage. The modified conventional formulas for the I_DS_-V_DS_ characteristic curves are first introduced not only for MOSFET and FinFET but also for IGBT’s internal triggered current. I_DS_ is due to moving charged carriers (mobility (μ) is larger than zero), as the gate bias is larger than the threshold voltage, which is derived in Section 2. Somehow, the kink effects are introduced to address the heat from the accelerated charges. The heat or the radiation causes the vibrations in the lattice-forming phonons and, thus, slows down the speed of carriers. The electric characteristic curves of the three transistors are fitted to determine the three basic parameters (k_N_, V_th_, λ). Moreover, the algorithm of the diode current–voltage characteristic curve combined with the gated control algorithm is used to characterize the measured data of the IGBT in Section 3. Those formulas are useful for fitting as-measured curves. The fitting results are analyzed and discussed in Section 4 and, thus, inform the conclusions in Section 5.

## 2. Threshold Voltage and the Strong Inversion Layer

**Figure 2:**
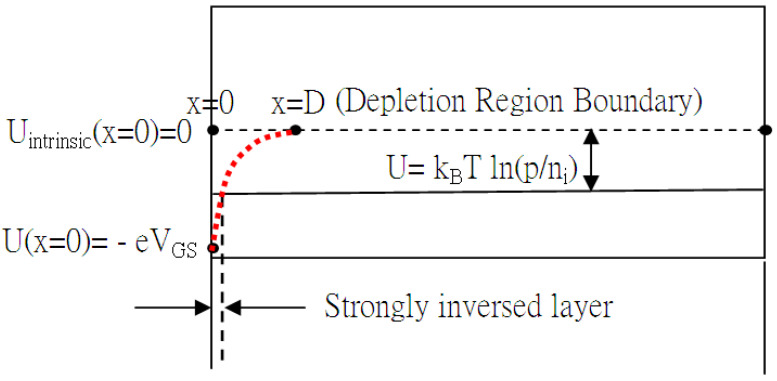
Constant p-type Fermi energy and silicon energy gap with intrinsic Fermi energy that may bend down as positive bias is applied to gate.

The silicon energy band is conceptually graphed in Figure 2, where U(x) = –eV(x). Suppose that the depth of the depletion region is D (where there exist no carriers in the beginning, except within the strong inversion layer), with the applied bias, V_GS_, and the boundary condition is set to V(D) = 0. The voltage function V(x) is given in Equation (1), and the threshold voltage is expressed in Equation (2) as follows:

(1) $$V(x)=\frac{pe}{\varepsilon_{Si}}(\frac{1}{2}x^{2}-Dx)+\frac{1}{2}\frac{pe}{\varepsilon_{Si}}D^{2}$$

As x = D, D is solved as follows:

$$D=\sqrt{\frac{2\varepsilon_{Si}}{pe}V_{GS}}$$

Also, for $V_{GS}\geq 2\left|\Phi_{p}\right|=2\frac{k_{B}T}{e}\ln(\frac{p}{n_{i}})$, the corresponding V_th_ and energy function, U(x), are listed in Equations (2) and (3), respectively. In order to solve the thickness, d, of the strong inversion layer, V(x) is set to $\left|\Phi_{p}\right|=\frac{k_{B}T}{e}\ln(\frac{p}{n_{i}})$. And *d* is solved and found in Equation (4). Of course, the zeroth-order threshold voltage V_tho_ at $V_{GS}=2\left|\Phi_{p}\right|=2\frac{k_{B}T}{e}\ln(\frac{p}{n_{i}})$ is as follows:

(2) $$V_{th}=(-0.56-\left|\Phi_{p}\right|{)}_{FB}-\frac{Q_{ox}^{\prime }}{C_{ox}^{\prime }}+V_{GS}+\frac{Q_{dep}^{\prime }}{C_{ox}^{\prime }}$$

(3) $$U(x)=-eV(x)=-\frac{pe^{2}}{\varepsilon_{Si}}(\frac{1}{2}x^{2}-Dx)-eV_{GS}$$

(4) $$d=x_{strong\_inversion\_layer}=D(1-\sqrt{\frac{\Phi }{V_{GS}}})$$

Within d, there are carriers that move freely with certain mobility.

## 3. Current–Voltage Formulas Associated with Threshold Voltage

When the applied gate voltage is over the threshold voltage, the channel becomes conductive. This is because the inversion layer in the channel turns out to be the same type as the Source and the Drain. For example, the n-p-n underneath the gate of NMOSFET turns out to be n-n-n, which is conductive. The I_DS_ at V_GS_ > V_th_, thus, soars up as V_DS_ is increased. With the above algorithm and Ohm’s Law $\left(J_{DS}=nev_{N}=ne\mu_{N}E_{DS}=\sigma_{N}E_{DS}\right)$, I_DS_-V_DS_-modified conventional formulas are derived and characterized in Equation (5), which may be used to fit the as-measured I_DS_-V_DS_ curves at different V_GS_. With the minimum δ (root mean square) in Equation (6), the extracted parameters (k_N_, V_th_, λ) are useful for further analyzing electrical performance if needed. k_N_ is related to the sizes of the transistor, the oxide capacitor, and the mobility (μ_N_) of the carriers. Also, there exists a term which is associated with the leakage current, i.e., $\lambda =\frac{1}{\left|V_{A}\right|}$, and Early voltage (V_A_).

(5) $$\left\{\begin{array}{l} I_{DS}(triode)=k_{N}[(V_{GS}-V_{th})V_{DS}-\frac{V_{DS}^{2}}{2}](1+\lambda V_{DS})\\ I_{DS}(saturation)=k_{N}[\frac{(V_{GS}-V_{th}{)}^{2}}{2}](1+\lambda V_{DS}) \end{array}\right.$$

(6) $$where\;k_{N}=\frac{{C_{ox}}^{\left(1\right)}W_{eff}\mu_{N}}{L_{o}}.\;\delta =\sqrt{\frac{\sum_{N}(I_{fitting}-I_{measured}{)}^{2}}{N}}$$

Moreover, kink effects are introduced as in Equation (7), with extra parameters (α, β, χ)_kink_. Kink effects may happen simply because the most accelerated carriers in the triode region emit a lot of photons running ahead, vibrating the lattice, causing obstacles, and reducing the speed of the carriers themselves.

(7) $$\left\{\begin{array}{l} I_{DS}(triode)=k_{N}[(V_{GS}-V_{th})V_{DS}-\frac{V_{DS}^{2}}{2}](1+\lambda V_{DS})\\ -\alpha \exp[-\beta (V_{\mathrm{DS}}-\chi {)}^{2}]\\ I_{DS}(saturation)=k_{N}[\frac{(V_{GS}-V_{th}{)}^{2}}{2}](1+\lambda V_{DS})\\ -\alpha \exp[-\beta (V_{\mathrm{DS}}-\chi {)}^{2}] \end{array}\right.\;\;where\;(\chi ,\alpha )\equiv (V_{DS\_kink},I_{DS\_kink}).$$

In addition, the IGBT is very structurally linked to the Bipolar Junction Transistors (BJTs), which are basically two diode combinations. The forward voltage applied to one of the diodes triggers the Base–Emitter current, which may flow through the Base to the Collector. This is because the Base cannot absorb all the carriers coming from the Emitter in time. So, the forward voltage (V_BE_) applied to the diode results in a Collector–Emitter current, which becomes somewhat saturated as V_BE_ or, thus, I_BE_ is finite, even though V_CE_ keeps increasing. In the IGBT, the hidden BJT devices are found in p-n-p or n-p-n forms everywhere, which are ignited by the Base current. The supplying Base current is apparently initially supplied by isolated gate-controlled ones, which is MOSFET through the double-diffusion process. This I_CE_ may be expressed as follows:

$$I_{CE}=I_{o}[\exp(\frac{eV_{BE}}{k_{B}T})-1]=I_{o}[\exp(\frac{\eta I_{BE}}{k_{B}T})-1]$$

What if I_BE_ is provided by the MOSFET (insulated gate, IG in IGBT)? With

$$\xi \equiv \frac{\eta }{k_{B}T},$$

the whole generated current is expressed as follows:

(8) $$I_{CE}=I_{o}[\exp(\frac{\eta I_{IG}}{k_{B}T})-1]=I_{o}[\exp(\xi I_{DS})-1]$$

(9) $$\begin{array}{l} I_{CE}=I_{o}[\exp(\frac{\eta I_{IG}}{k_{B}T})-1]\mp \alpha \exp[-\beta (V_{\mathrm{DS}}-\chi {)}^{2}]\\ =I_{o}[\exp(\xi I_{DS})-1]\mp \alpha \exp[-\beta (V_{\mathrm{DS}}-\chi {)}^{2}] \end{array}$$

Note that I_DS_ in Equation (8) actually obeys the formulas in Equation (5). Nevertheless, in order to address kink effects and bulk effects, Equation (9) is added with a minus sign to address kink effects and a plus sign to address bulk effects. The differences are explained as follows: (1) Hot carriers corresponding to higher speeds and their variations would generate radiation that causes more phonon scattering or obstacles than absorbing heat/EM radiation from the bulk around. (2) Cool carriers corresponding to lower speeds and their variations would generate radiation that causes less phonon scattering or obstacles than absorbing heat from the bulk around.

## 4. Results and Discussion

**Figure 3:**
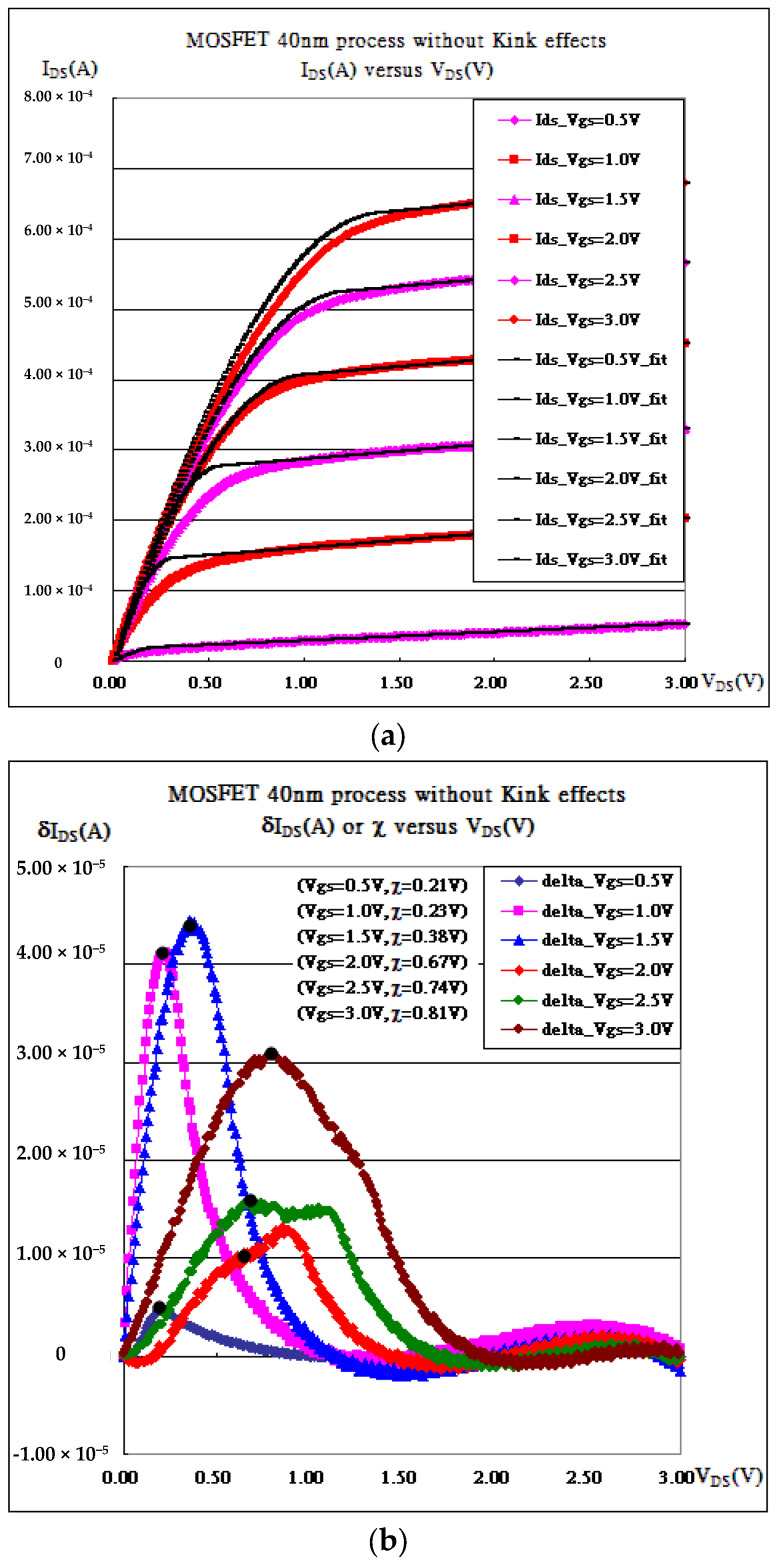

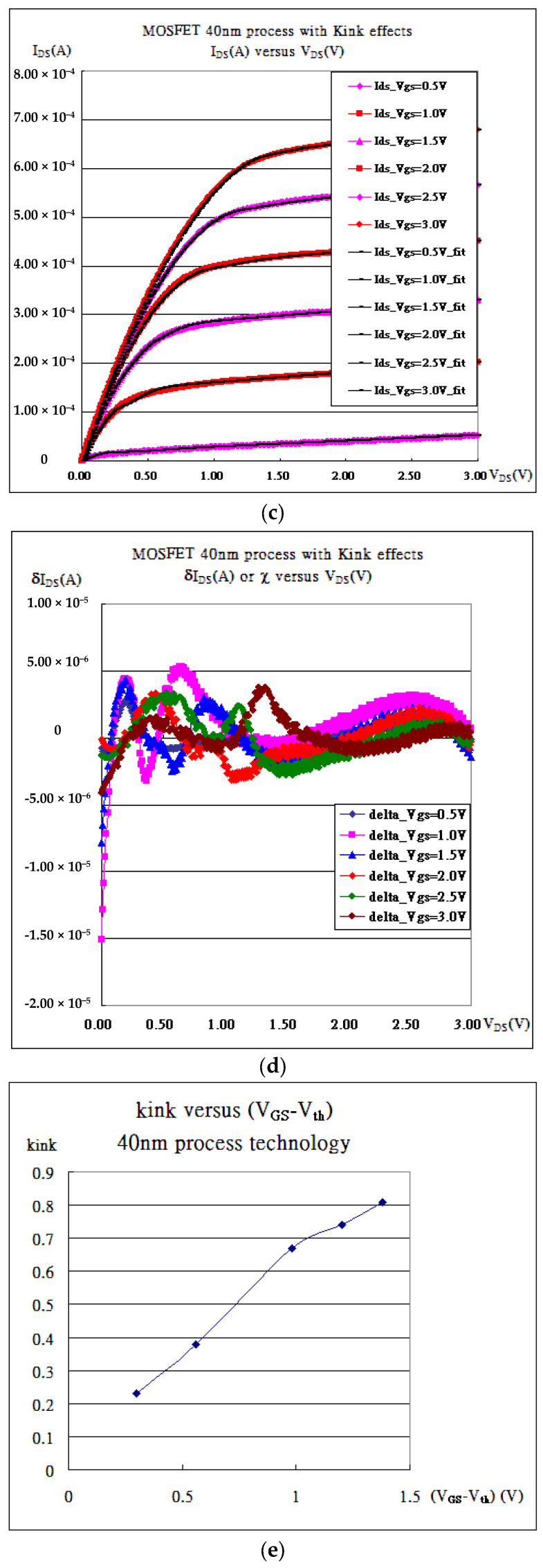
MOSFET 40 nm process. (**a**) I_DS_-V_DS_ and fitting without considering kink effects. (**b**) Deviation between fitting data and measured data without considering kink effects and with RMS (V_GS_ = 0.5 V, V_th_ = 0.28 V) = 1.38 × 10^−6^, RMS (V_GS_ = 1.0 V, V_th_ = 0.7 V) = 1.21 × 10^−5^, RMS (V_GS_ = 1.5 V, V_th_ = 0.94 V) = 1.56 × 10^−5^ , RMS (V_GS_ = 2.0 V, V_th_ = 1.02 V) = 5.08 × 10^−6^, RMS (V_GS_ = 2.5 V, V_th_ = 1.30 V) = 7.78 × 10^−6^, and RMS (V_GS_ = 3.0 V, V_th_ = 1.62 V) = 1.54 × 10^−5^. (**c**) I_DS_-V_DS_ and fitting considering kink effects. (**d**) Deviation between fitting data and measured data considering kink effects and with RMS (V_GS_ = 0.5 V, V_th_ = 0.28 V) = 7.11 × 10^−7^, RMS (V_GS_ = 1.0 V, V_th_ = 0.7 V) = 2.70 × 10^−6^, RMS (V_GS_ = 1.5 V, V_th_ = 0.94 V) = 1.69 × 10^−6^ , RMS (V_GS_ = 2.0 V, V_th_ = 1.02 V) = 1.49 × 10^−6^, RMS (V_GS_ = 2.5 V, V_th_ = 1.30 V) = 1.55 × 10^−6^, and RMS (V_GS_ = 3.0 V, V_th_ = 1.62 V) = 1.26 × 10^−6^. (**e**) A strong correlation exists between kinks and (V_GS_-V_th_).

As presented in Figure 1(a1–a3) and Figure 3, the characteristic curves of the 40 nm planar MOSFET process are fitted without or with kink effects. Apparently, the root mean squares with kink effects always have smaller values than those without. That is because the two formulas in Equation (5) never take care of the radiation by accelerated charges, which may cause extra obstacles for carriers. Once the reductions in the kink effects due to the obstacles are taken into account, the fitting curves move much closer to the measured ones, as referred to in Figure 3d. Kink is always chosen to be the top of the hill of the deviation. But Figure 3b shows double hills at V_GS_ = 2.0 V and V_GS_ = 2.5 V such that the kink is difficult to define. They (kink_2.0 = 0.67 V, kink_2.5 = 0.74 V) and all extracted parameters are listed in Table 1. Nevertheless, a strong correlation exists between V_DS_ (kinks) and (V_GS_-V_th_), as shown in Figure 3e, where the kinks are demonstrated to be reasonable.

**Figure 4:**
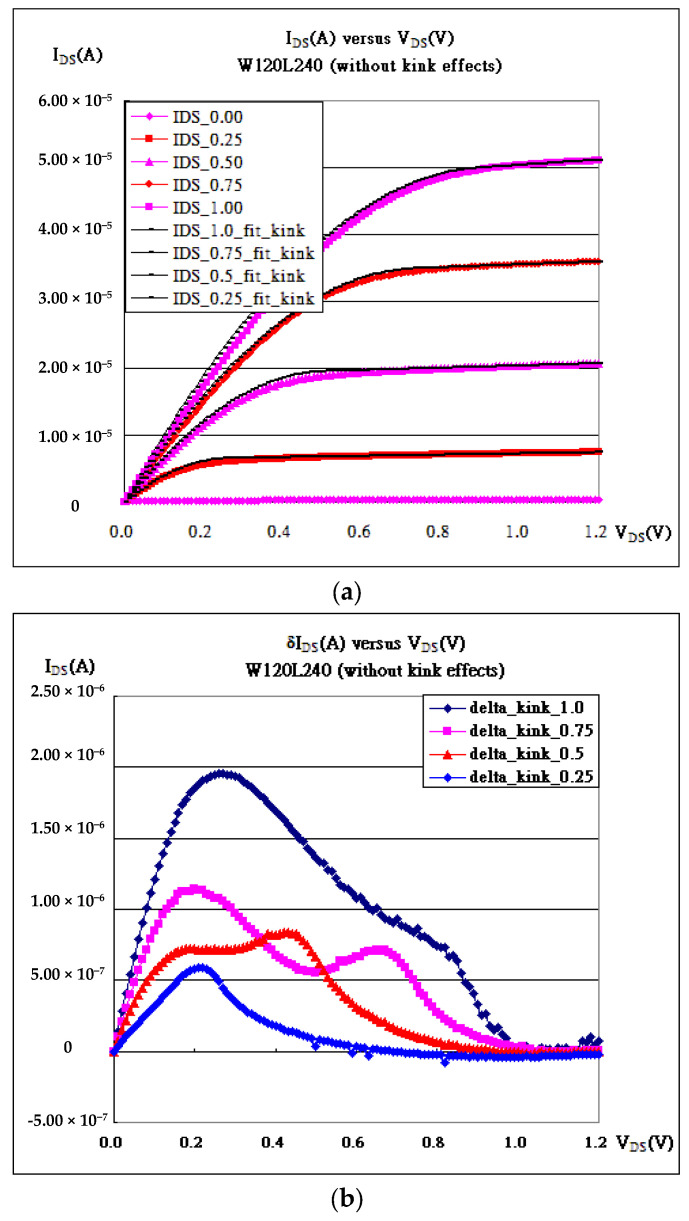

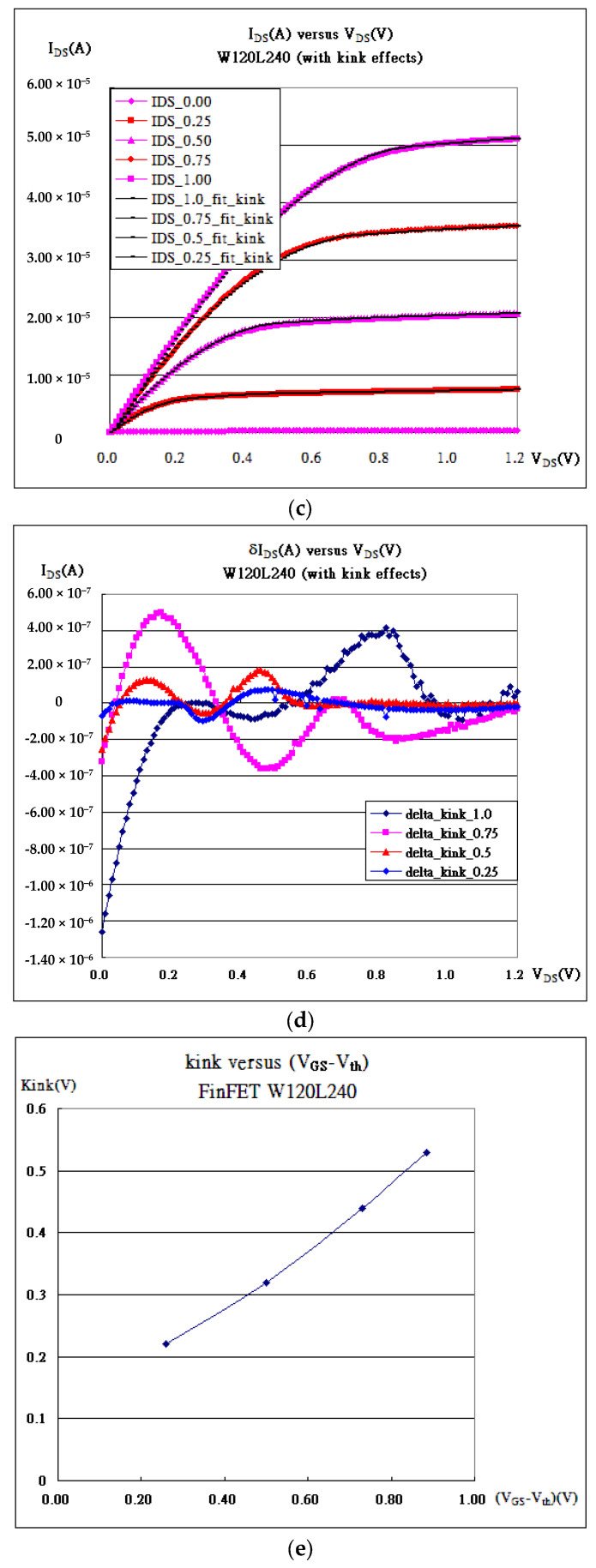
W120 L240 nm FinFET process. (**a**) I_DS_-V_DS_ and fitting without considering kink effects. (**b**) Deviation between fitting data and measured data without considering kink effects and with RMS (V_GS_ = 0.25 V, V_th_ = 0.0 V) = 2.25 × 10^−7^, RMS (V_GS_ = 0.5 V, V_th_ = 0.0 V) = 4.67 × 10^−7^, RMS (V_GS_ = 0.75 V, V_th_ = 0.0 V) = 6.22 × 10^−7^, and RMS (V_GS_ = 1.0 V, V_th_ = 0.1 V) = 1.15 × 10^−6^. (**c**) I_DS_-V_DS_ and fitting considering kink effects. (**d**) Deviation between fitting data and measured data considering kink effects and with RMS (V_GS_ = 0.25 V) = 3.99 × 10^−8^ (V_th_ = 0.0 V), RMS (V_GS_ = 0.5 V) = 6.97 × 10^−8^ (V_th_ = 0.0 V), RMS (V_GS_ = 0.75 V) = 2.34 × 10^−7^ (V_th_ = 0.0 V), and RMS (V_GS_ = 1.0 V) = 3.04 × 10^−7^ (V_th_ = 0.1 V). (**e**) A strong correlation exists between kinks and (V_GS_-V_th_).

In Figure 1b and Figure 4, the characteristic curves of a FinFET with fin width = 120 nm and channel length = 240 nm are fitted without or with kink effects. Apparently, the root mean squares with kink effects always have smaller values than those without, as shown in Figure 4d. Kink is always chosen to be the top of the hill of the deviation. But Figure 4b shows double hills because of the 3D structure of the FinFET with double walls. The kinks are always chosen to be the average of the two hills, as listed in Table 2. Surely, kink may not be arbitrarily chosen, because kink (V_DS_kink_) is always chosen to be a little less than (V_GS_-V_th_). In addition, both are closely and strongly correlated, referring to Figure 3e and Figure 4e.

**Figure 5:**
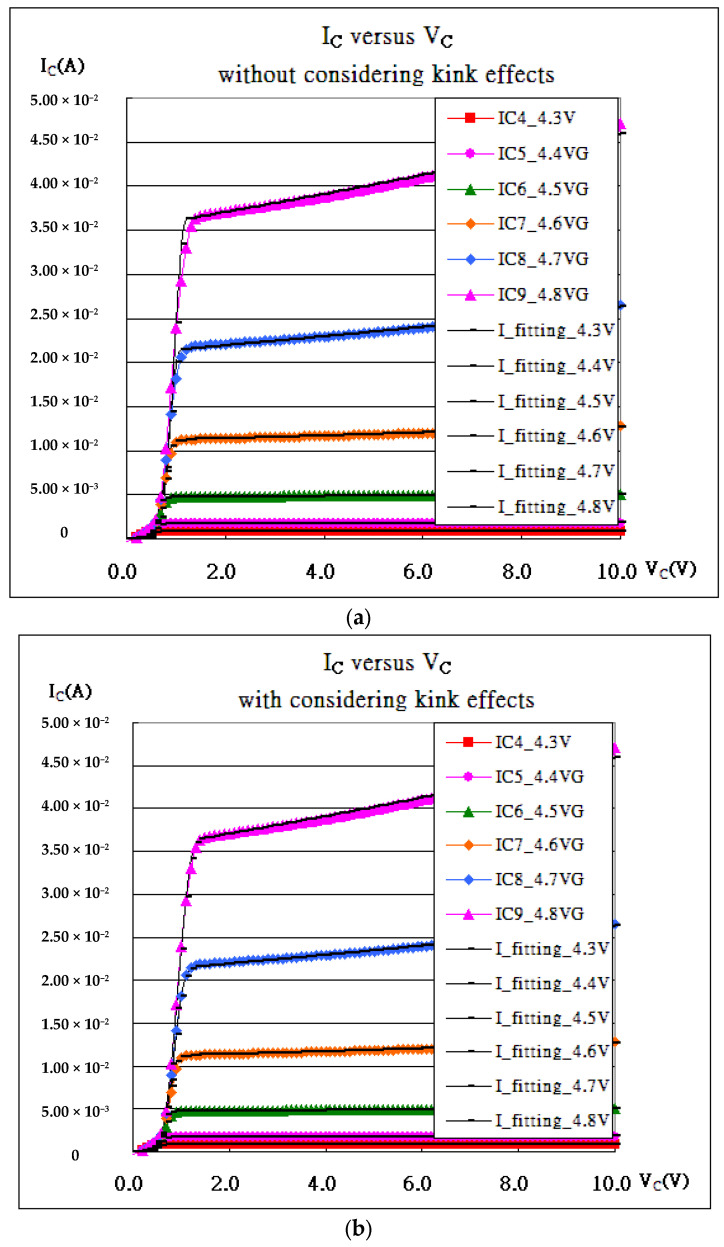

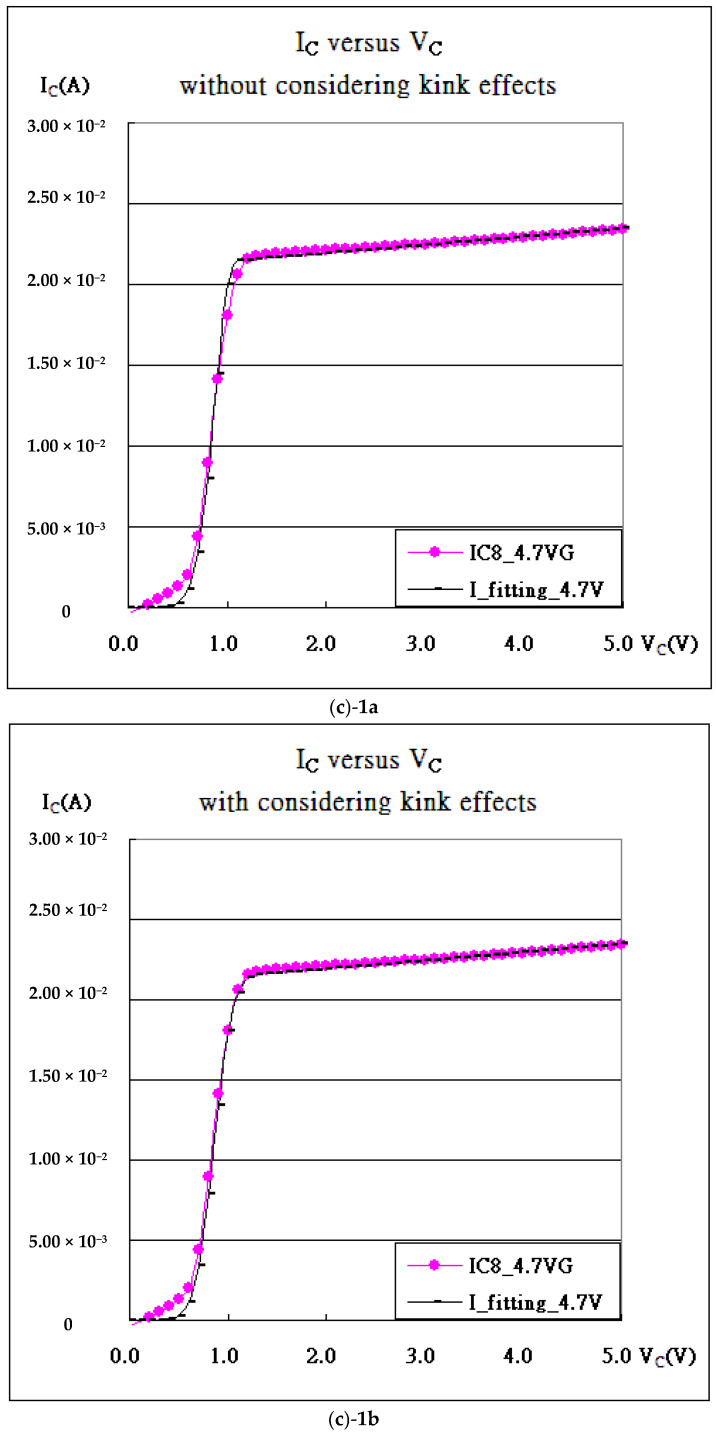

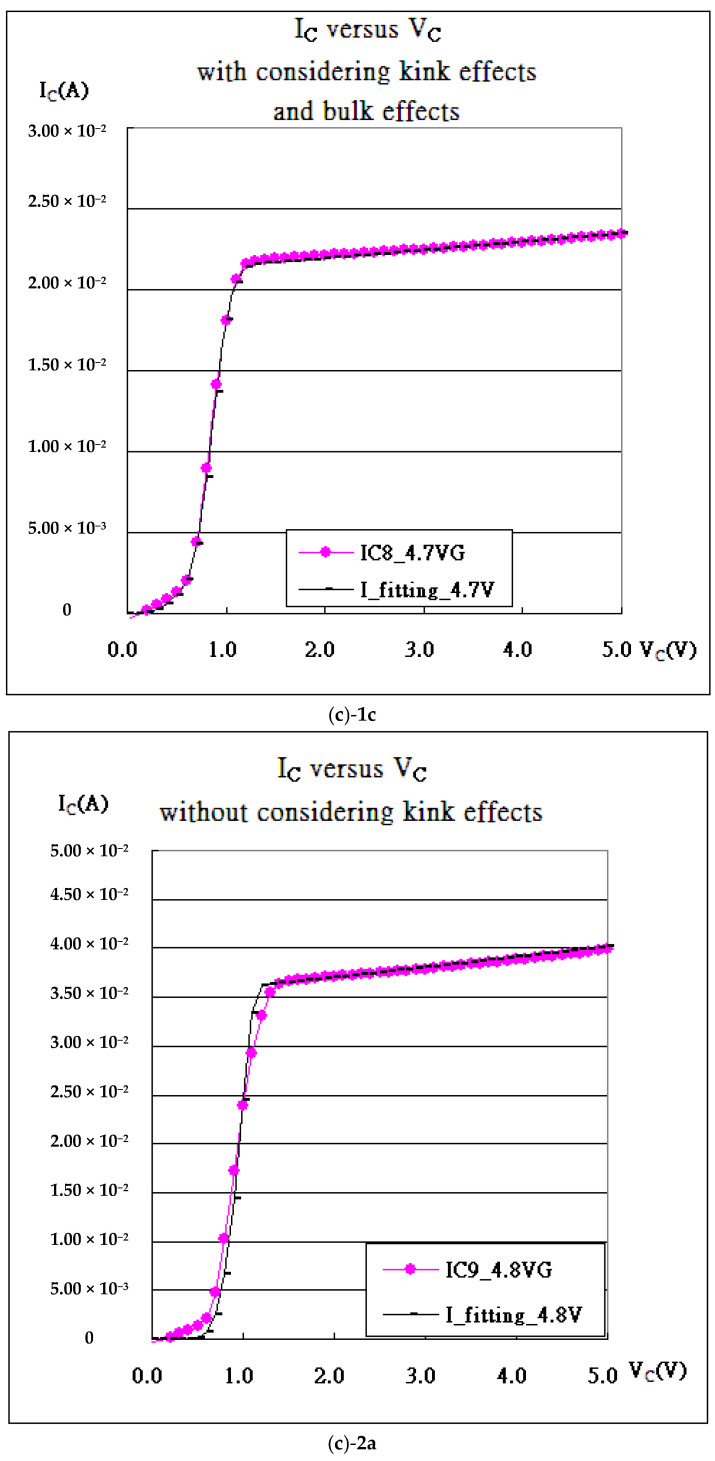

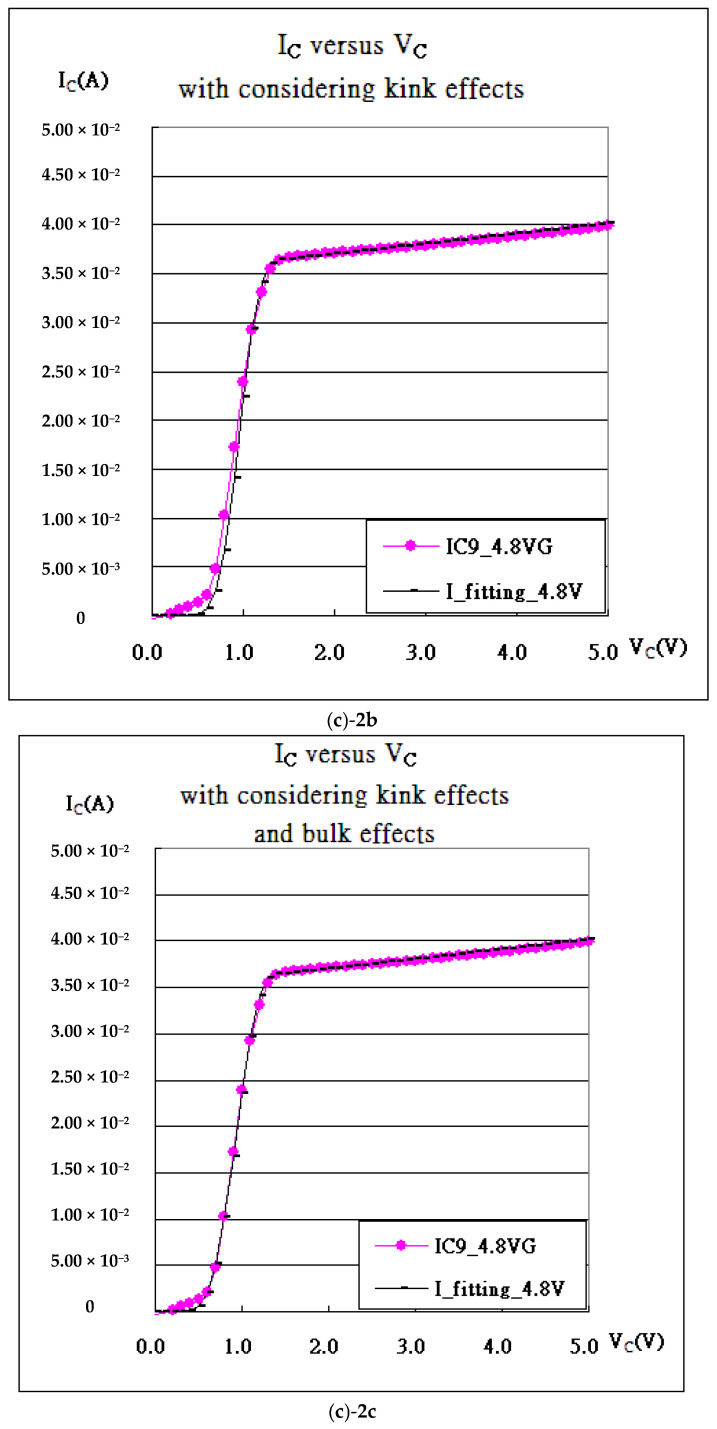
(**a**) The characteristic curves, I_CE_-V_CE_, and their fitting for IGBT in Equation (8). (**b**) The characteristic curves, I_CE_-V_CE_, and their fitting for IGBT considering kink effects. (**c**-**1a**) V_G_ = 4.7 V without considering kink effects, (**c**-**1b**) V_G_ = 4.7 V considering kink effects, (**c**-**1c**) V_G_ = 4.7 V considering kink effects and bulk effects, (**c**-**2a**) V_G_ = 4.8 V without considering kink effects, (**c**-**2b**) V_G_ = 4.8 V considering kink effects, and (**c**-**2c**) V_G_ = 4.8 V considering kink effects and bulk effects.

As shown in Figure 1c and Figure 5, the fitting in Figure 5b is always superior to that in Figure 5a for the kink effects (the radiation by accelerated charges), and bulk effects join the fitting. Bulk effects, taking the same form as kink effects but with a positive sign, address the heat coming from the bulk and accelerating the carriers. Figure 5(c-1a–1c) (V_GS_ = 4.7 V) and Figure 5(c-2a–2c) (V_GS_ = 4.8 V) show the extra improvements in the fitting as referred. All extracted internal parameters are listed in Table 3.

**Figure 6:**
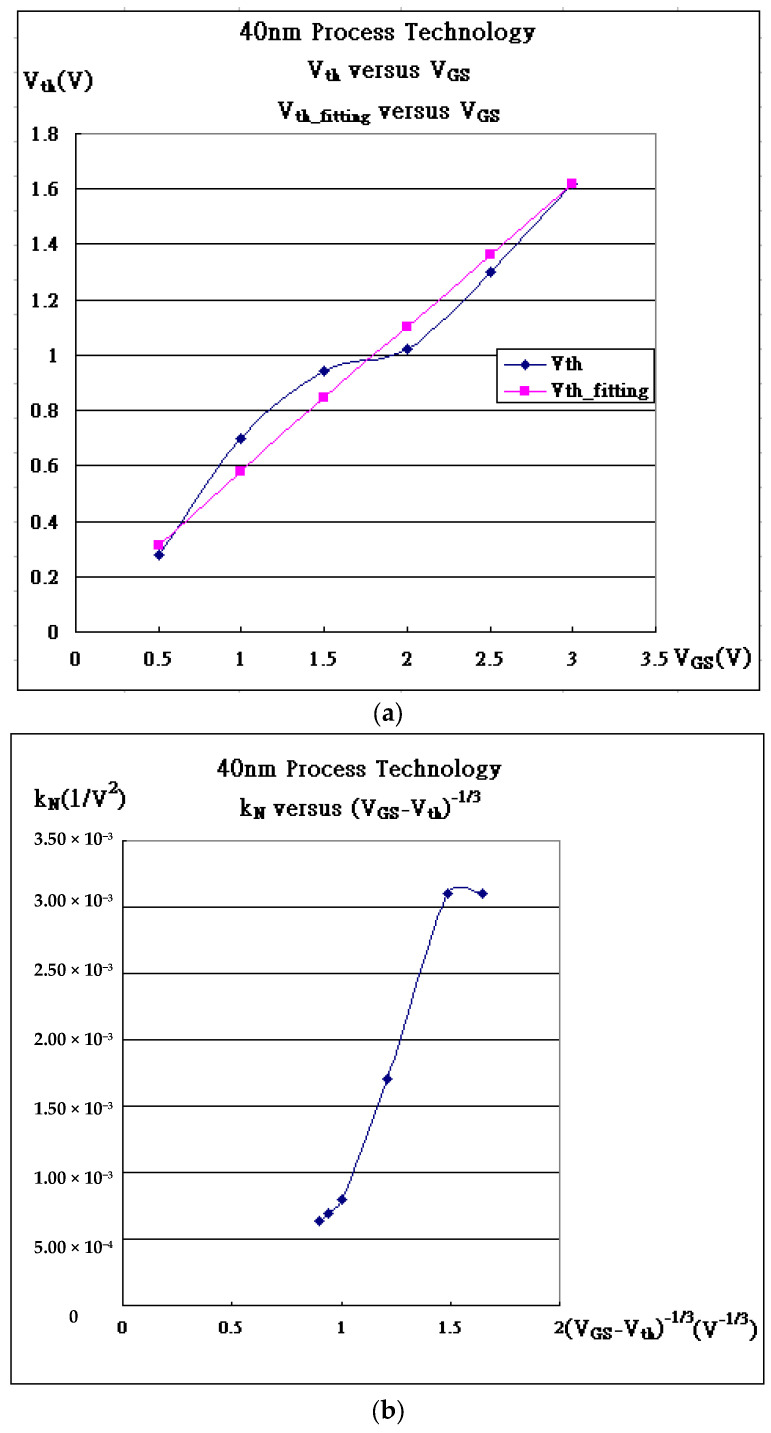
40 nm MOSFET process. (**a**) The fitting V_th_ ($V_{th0}=0.0+0.481V_{GS}+0.1\sqrt{V_{GS}}$). (**b**) k_N_ versus (V_GS_ − V_th_)^−1/3^.

In Table 1, V_th_ plotted against V_GS_ may be determined from the V_th_ formula in Equation (2), where V_tho_ can be expressed as V_GS_ = |2Φ|. The fitting of V_th_ ($V_{th0}=0.0+0.481V_{GS}+0.1\sqrt{V_{GS}}$) is plotted in Figure 6a, which provides the approximate concentration of p(1/m^3^) = 1.62 × 1022(1/m^3^) and where the nitrided gate-oxide thickness is 60 Å with dielectric constantκ = 5.2. If the concentration, p, is substituted into $\left|\Phi_{p}|=\frac{k_{B}T}{e}\ln(\frac{p}{n_{i}})=3.58\times 10^{-1}(V\right)$ at room temperature, the corresponding strong inversion layer in Equation (4) is then 310.7 Å thick at V_GS_ = 0.5 V. Furthermore, the mobility (μ) is proportional to (V_GS_ − V_th_)^−1/3^, as referred to in Figure 6b [22]. Somehow, the V_th_-fitting inconsistency in Figure 6a is correlated with the carrier mobility, as compared to Figure 6b. There are two linear sets, including (V_GS_ = 0.5, 1.0, 1.5 V) and (V_GS_ = 2.0, 2.5, 3.0 V), that we have to compromise. The mobility at V_GS_ = 0.5 V is surprisingly small, probably because of quantum confinement making carriers move more clumsily.

**Figure 7:**
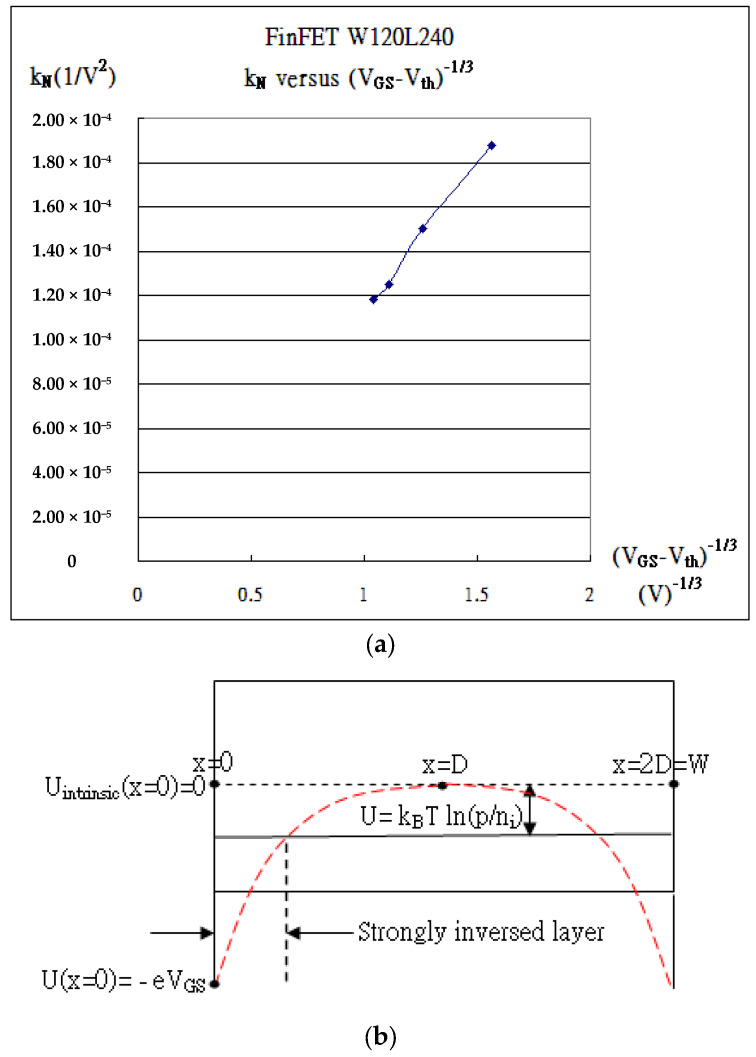
W120 L240 FinFET process. (**a**) k_N_ versus (V_GS_ − V_th_)^−1/3^. (**b**) FinFET is supposed to be with the fin width 2D = 120 nm at V_GS_ = 1 V.

In Table 2, the mobility (μ) is proportional to (V_GS_ − V_th_)^−1/3^, referred to as k_N_ versus (V_GS_ − V_th_)^−1/3^ in Figure 7a [22]. Moreover, the farthest depletion region D may be found in Equation (1) on one side at V_GS_ = 1.0 V. The approximate concentration of p(1/m^3^) = 3.66 × 10^23^(1/m^3^), which may be identified as 2D, is set to 120 nm. And the corresponding strong inversion layer in Equation (4) is then 203 Å thick at V_GS_ = 1.0 V.

**Figure 8:**
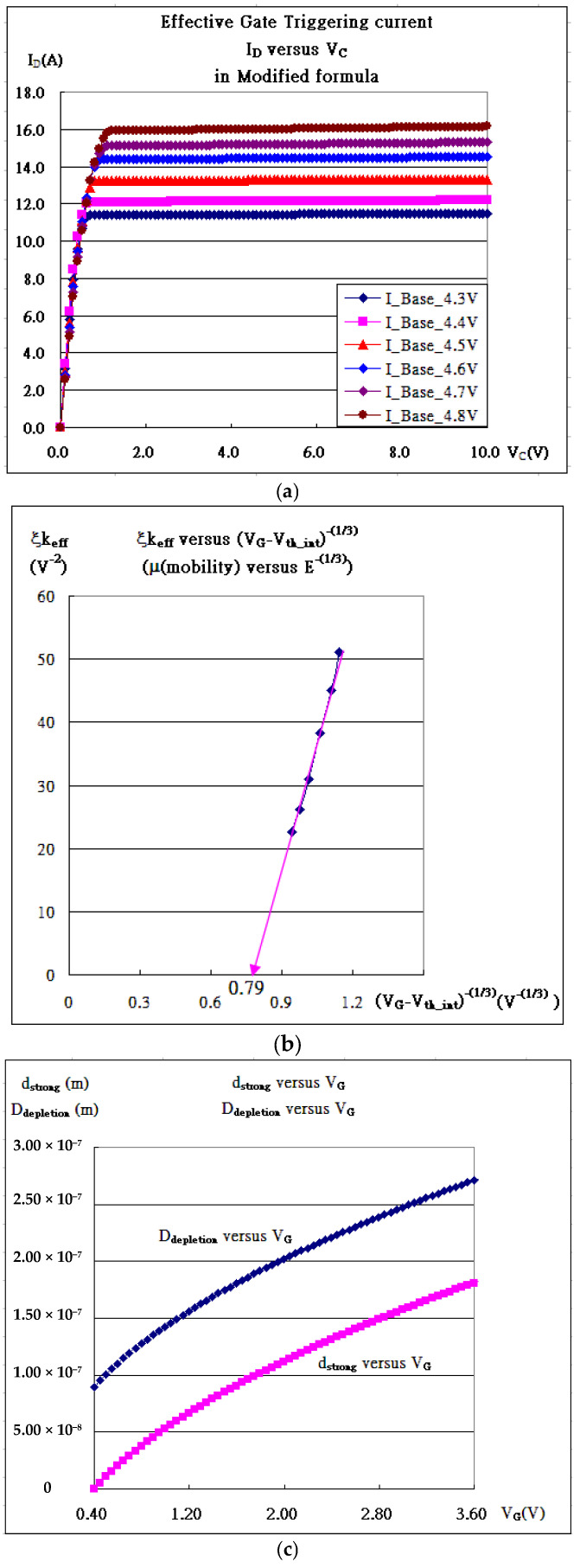
IGBT. (**a**) The internal I_DS_ versus V_DS_. (**b**) k_N_ versus (V_GS_ − V_th_)^−1/3^. (**c**) The depletion region thickness D and the strong inversion layer d with V_G_ less than V_tho_ (3.6 V).

In Table 3, the insulated gate behaves like the gate of MOSFET, and the internal characteristic curves are drawn in Figure 8a. All the internal extraction parameters are listed. Notice that the triggering current associated with the mobility (μ) in ξk_eff_ is electrical field E^−1/3^-dependent [22] or (V_GS_ − V_th_)^−1/3^-dependent, as shown in Figure 8b. The line linearly intercepts the (V_GS_ − V_th_)^−1/3^-axis at 0.79, which corresponds to V_GS_ = 5.55 V and μ → 0. Somehow, the mobility impossibly approaches zero, but there exists viscosity at the oxide interface, causing more obstacles in the movement of carriers as the applied V_GS_ is becoming higher. In addition, the current is due to the strong inversion layer induced by the applied bias to the gate, as this bias is slightly over the internal threshold voltage. The average internal threshold voltage in Table 3 is determined to be about the same value, V_tho_ = 3.6411 V,

$$V_{th0}=-0.56+|\Phi_{p}|+\frac{\sqrt{2\varepsilon_{Si}pe(|2\Phi_{p}|)}}{C_{ox}}$$

which gives the double-diffusion concentration, p = 6.45 × 10^22^/m^3^, from Equation (2), as Q_ox_ is ignored and the thickness of the oxide is set to 1000 Å. With p, the thickness of the strong inversion layer can be calculated from Equation (4), which gives d = 1830 Å at V_GS_ = 3.64 V. On the other hand, even though the strongly inverted layer is present for the conduction of the current at V_GS_ less than 3.64 V, as shown in Figure 2 and Figure 8c, the viscosity of silicon totally blocks the conduction current until V_GS_ is larger than 3.64 V. The viscosity reflects mobility degradation, interfacial scattering, or quantum confinement; this needs more elaborate work in the near future. Intriguingly, the existing trapping would stop carriers from moving unless the applied gate bias is over the threshold voltage. The quantum confinement addresses the quantum well, which traps the carriers. The thickness of the strong inversion layer, d = 1.80 × 10^−7^ m at V_GS_ = 3.6 V, as shown in Figure 8c, corresponds to the momentum, *p_3.6v_ = h/d* = 3.68 × 10^−27^ m/s (Uncertainty Principle), as the minimum one of the electron that becomes conductive. From I–V in the IGBT, the momentum of the electron carrier is found to be *p_e_ = m_e_v* = 6.59 × 10^−27^ (m/s), where *v = J/ne = I/(Ane) = I/(dWne)* with I = 1.0 × 10^−6^ (A), n = 6.45 × 10^22^/m^3^, W_effective_ = 4 × 12 × 10^−6^ × 10000(m), and d = 1.80 × 10^−7^ m. Both calculations are about the same order and quite consistent to support *p_e_ ≥ p_3.6v_.*

Moreover, moving carriers, including electrons and holes, flow along the channel, carrying electrical power or signals. Somehow, the carriers promptly become accelerated and shortly reach a higher speed due to an increasing V_DS_. Suppose that the frictional force opposing the electrical force is proportional to the power of N of the speed of every single carrier. For example, the total force F on the electron of m_e_ (mass of electron) is characterized as follows:

(10) $$F_{DS}=-eE_{DS}+\varsigma v^{N}$$

where ζ is a viscosity coefficient and E_DS_ is the electrical field coming from the applied bias along the channel. Before electrons reach the maximum terminal speed,

(11) $$\frac{dv}{dl}v=-\frac{e}{m_{e}}E_{DS}+\frac{\varsigma }{m_{e}}v^{N}$$

where *l* is the extremely short traverse distance length that the electron travels as *E_DS_* is suddenly applied. So,

(12) $$l=\int_{0}^{v_{term}}\frac{m_{e}vdv}{-eE_{DS}+\varsigma v^{N}}=\int_{0}^{v_{term}}f(v)dv$$

where

(13) $$f(v)=\frac{m_{e}v}{-eE_{DS}+\varsigma v^{N}}$$

From Equation (12), *l* is mainly determined by the denominator approaching to zero with the applied electrical field *E_DS_ = V_DS_/L*, as stated below:

(14) $$-eE_{DS}+\varsigma v^{N}≅0$$

and

(15) $$v=\sqrt[{N}]{\frac{eE_{DS}}{\varsigma }}\overset{N=1}{\to }v=\frac{e}{\varsigma }E_{DS}$$

which gives the information that the mobility in the conventional *I_DS_ (V_DS_, V_GS_)* formula in Equation (5) is constant at a certain fixed gate bias only when *N = 1*. And the mobility is then expressed as follows:

(16) $$\mu =\frac{e}{\varsigma }$$

which verifies that the constant mobility at a certain fixed gate bias is inversely proportional to the viscosity coefficient.

Comparing the three tables, the extracted threshold voltages play important roles depending on the structures and the sizes. The 40 nm process uses strain technology that promotes electrical performance. Nevertheless, the variations in the extracted threshold voltages reveal some kind of sensitive internal energy band structure. Furthermore, FinFET exposes the negative threshold voltages at V_GS_ = 1.0 V, which might be associated with sizes or quantum effects. For the power discrete devices, the empirical internal threshold voltages show stable constants, which can be used to evaluate the external threshold voltages (around 4.3 V). Of course, kn refers to the comparably controllable sizes of the transistor and the intriguing mobility, which may availably provide more insight into the underlying physics.

As for the current gain in the IGBT, h_21_ is defined and expressed as follows:

(17) $$\begin{array}{l} h_{21}\equiv (\frac{\partial I_{CE}}{\partial I_{Base}}{)}_{V_{GE}=fixed}=\tau (\frac{\partial I_{CE}}{\partial V_{CE}}{)}_{V_{GE}=fixed}\\ =\tau I_{o}\xi \exp[\xi I_{C}](\frac{\partial I_{C}}{\partial V_{C}}{)}_{V_{GE}=fixed} \end{array}$$

where the feature of kink may be closely associated with the term (${\left(\frac{\partial I_{C}}{\partial V_{C}}\right)}_{V_{GE}=fixed}$), as referred to in Figure 1d and Figure 5, and so is h_21_. The kink is supposed to disappear as the temperature is raised due to the exponential term in Equation (17). Other sorts of kinks might be blurred out due to temperature effects as well [2].

## 5. Conclusions

In this paper, (1) three kinds of gated transistors (MOSFET, FinFET, IGBT) are presented, which begin to flow current as a gate is applied with biases over the threshold voltage. The inversion layer, d, and the farthest depletion thickness, D, are formulated with the known concentrations p(1/m^3^), which may be calculated in three different ways. (2) As-measured I_DS_-V_DS_ curves of the three kinds of transistors are fitted with modified formulas in Equations (5), (7), (8), and (9). And the fittings are even more promising with kink effects and bulk effects. For one thing, the most accelerated carriers radiate photons, causing phonons to disturb themselves, become obstacles, and reduce their speed, especially prior to the saturation regions. The other is that the cool carriers may gain bulk radiation and then become accelerated, especially and apparently on the IGBT (3) The thicknesses of the strong inversion layer are calculated and are reasonable. (4) k_N_ is defined as (μWC_ox_^(1)^/L_o_) and, therefore, is proportional to μ if W, C_ox_^(1)^, and L_o_ are fixed. The mobility may be found in three different ways as follows: (a) With different gate biases larger than the threshold voltage, mobility (μ) is linearly dependent of E^(−1/3)^ and, thus, proportional to (V_GS_ − V_th_)^(−1/3)^). (b) With gate biases larger than (k_B_T/e)ln(p/n_i_) but less than the threshold voltage, μ = 0 because the existing trapping would stop carriers from moving unless the applied gate bias is over the threshold voltage. The quantum confinement, thus, addresses the quantum well, which traps the carriers. (c) Mobility shall be constant as applied with a fixed gate bias, which is larger than the threshold voltage. The mobility is proven to be inversely proportional to the viscosity (viscosity = ζ, and $\mu =\frac{e}{\varsigma }$).

The present work proposes a feasible way to further improve the fitting of I_DS_-V_DS_ characteristic curves using the modified conventional formulas, which are associated with gated transistors. The gated transistors include MOSFET, FinFET, and IGBT. The I_DS_-V_DS_ curve in the triode region has a maximum slope of $G_{m}={\left.\frac{\partial I_{DS}}{\partial V_{DS}}\right|}_{V_{GS}=fixed}$, which is prior to the turning point to the saturation region, as seen in Figure 1d. The whole channel is inevitably influenced by the emitting photons due to the speed variation in the charged carriers. The emitting photons most likely interact with the lattice and cause extra vibrations of the lattice, which are quantized as phonons. Phonons, of course, physically reduce the speeds of carriers. G_m_ looks like a hill, and it is where the kink is located. The kink is observed in the trans-conductance (g_m_, S_22_) and the current gain (h_21_), which may be flat as the temperature is increased up to, e.g., 200 °C or even as the applied gate/drain bias is varied [6]. In a word, there shall be no gap among those various kinks.

This promising mechanism gives much confidence to the exploration of devices using 20 nm process technology, which will expose more of the quantum world. Nevertheless, this paper studies the devices presumably at 25 °C. For large sizes, such as the 2μm process, the temperature effects show no apparent differences. But for transistors of small sizes, such as 90 nm or below, the temperature effects show significant differences in terms of the threshold voltage, mobility, and kink effects, so the copper conduction, CoWoS (Chip on Wafer on Substrate), and buried tunnels with the functions of the total reflection of infrared rays are found to be very important on the package level.

## Figures and Tables

**Table 1:** Transistors using 0.040 μm process technology with where kink is located.

Gate Bias(V)	k_N_ (A/V^2^)	V_th_fit_ (V)	λ (1/V)	Kink (V_DS_)
V_GS_ = 0.50 V	6.50 × 10^−4^	0.28	0.738	0.21
V_GS_ = 1.00 V	3.10 × 10^−3^	0.70	0.151	0.23
V_GS_ = 1.50 V	1.70 × 10^−3^	0.94	0.083	0.38
V_GS_ = 2.00 V	8.00 × 10^−4^	1.02	0.059	0.67
V_GS_ = 2.50 V	6.90 × 10^−4^	1.30	0.047	0.74
V_GS_ = 3.00 V	6.30 × 10^−4^	1.62	0.043	0.81

**Table 2:** Transistors using W120 L240 FinFET process technology with where kink is located.

Gate Bias (V)	k_N_ (A/V^2^)	V_th_fit_ (V)	λ (1/V)	Kink (V_DS_)
V_GS_ = 0.25 V	1.88 × 10^−4^	−0.01	0.15	0.22
V_GS_ = 0.50 V	1.50 × 10^−4^	0.00	0.09	0.32
V_GS_ = 0.75 V	1.25 × 10^−4^	0.02	0.07	0.44
V_GS_ = 1.00 V	1.18 × 10^−4^	0.11	0.08	0.53

**Table 3:** IGBT with V_G_ slightly over internal threshold voltage.

Gate Bias (V)	ξk_N_ (1/V^2^)	V_th_fit_ (V)	λ (1/V)	I_o_ (A)
V_GS_ = 4.3 V	51.142	3.63257	0.00065	9.29 × 10^−9^
V_GS_ = 4.4 V	45.023	3.66703	0.00068	9.29 × 10^−9^
V_GS_ = 4.5 V	38.201	3.66687	0.00070	8.35 × 10^−9^
V_GS_ = 4.6 V	31.062	3.63793	0.00101	6.31 × 10^−9^
V_GS_ = 4.7 V	26.195	3.62690	0.00151	5.90 × 10^−9^
V_GS_ = 4.8 V	22.625	3.61557	0.00170	4.50 × 10^−9^

## Data Availability

The original contributions presented in this study are included in the article. Further inquiries can be directed to the corresponding author.
